# Epigenetic Silencing of miR-338-3p Contributes to Tumorigenicity in Gastric Cancer by Targeting SSX2IP

**DOI:** 10.1371/journal.pone.0066782

**Published:** 2013-06-24

**Authors:** Pu Li, Xuehua Chen, Liping Su, Chenglong Li, Qiaoming Zhi, Beiqin Yu, Hong Sheng, Junqing Wang, Runhua Feng, Qu Cai, Jianfang Li, Yingyan Yu, Min Yan, Bingya Liu, Zhenggang Zhu

**Affiliations:** Shanghai Key Laboratory of Gastric Neoplasms, Department of Surgery, Shanghai Institute of Digestive Surgery, Ruijin Hospital, School of Medicine, Shanghai Jiao Tong University, Shanghai, People’s Republic of China; University of Barcelona, Spain

## Abstract

MicroRNA has been recently recognized as playing a prominent role in tumorigenesis and metastasis. Here, we report that miR-338-3p was epigenetically silenced in gastric cancer, and its down-regulation was significantly correlated with gastric cancer clinicopathological features. Strikingly, restoring miR-338-3p expression in SGC-7901 gastric cancer cells inhibited proliferation, migration, invasion and tumorigenicity *in vitro* and *in vivo*, at least partly through inducing apoptosis. Furthermore, we demonstrate the oncogene SSX2IP is a target of miR-338-3p. We propose that miR-338-3p functions as a tumor suppressor in gastric cancer, and the methylation status of its CpG island could serve as a potential diagnostic marker for gastric cancer.

## Introduction

Gastric cancer is a malignancy with high incidence and the second leading cause of cancer death worldwide [1]. The early diagnostic rate of gastric cancer is low, and patients are usually difficult to be diagnosed until the cancer has developed into advanced stage or metastasis. Thus, it is of great significance to carry out in-depth study on the pathogenesis of gastric cancer and to look for new molecular makers and therapeutic targets.

MicroRNAs are small RNAs that regulate gene expression, and they are involved in a variety of biological signaling pathways [2]. Multiple studies have revealed significant difference of microRNAs expression profiling between tumor cells and cells derived from normal tissues, indicating that microRNAs have played important roles in tumorigenesis [3], [4]. Thus, MicroRNAs has recently become a hotspot of genetic diagnosis and treatment target as novel oncogenes or suppressor genes [5], [6]. Abnormal expression of microRNAs in tissues or serum is closely related to multiple human malignancies including gastric cancer [7], [8], and several down-regulated microRNAs in gastric cancer tissues have tumor suppressing functions. For instance, the Let-7 microRNA family was first found as a repressor of RAS, repressing RAS and c-myc expression at translational level [9]. The expression level of let-7a is significantly reduced in gastric tumors, while Ras protein is significantly higher in gastric tumor [10]. Moreover, miR-15b, miR-16 and miR-21 etc have been connected to the development and clinical diagnosis of gastric cancer [11], [12]. Our group has also reported the anticancer role of miR-126, miR-409-3p and miR-155 in gastric cancer [13]–[15].

MicroRNA microarray was performed to detect the miRNA profiles in gastric cancer, and we found that miR-338-3p was significantly down-regulated (unpublished data). miR-338-3p, located at chromosome 17q25.3 with a length of 22 nt, was first reported in prion-induced neurodegeneration as the expression of miR-338-3p is reduced in the brains of mice infected with mouse-adapted scrape [16]. miR-338-3p could regulate the mRNA level of its host gene Apoptosis-associated Tyrosine Kinase (AATK) in rat neurons [17]. On the other hand, miR-338-3p was up-regulated in patients with sporadic amyotrophic lateral sclerosis (sALS), another kind of nervous system lesions [18]. Importantly, a role of miR-338-3p in tumorigenesis has been previously suggested, as miR-338-3p is down regulated in rectal cancer [19], and is regulated by Hepatitis B virus X protein (HBx) to inhibit proliferation and invasion of hepatoma cells by binding to target genes CyclinD1 and smoothened in hepatocellular carcinoma [20]–[22]. However, the role of miR-338-3p in GC is still unknown.

In the present study, we further analyzed the down-regulation expression of miR-338-3p in GC tissues compared with patient-matched normal tissues, and determined that the expression level of miR-338-3p was closely correlated with the clinicopathologic variables of GC. Ectopic expression of miR-338-3p inhibits proliferation, invasion and metastasis of gastric cancer cells, reduces the tumorigenic ability of gastric cancer cells in nude mice, and promotes apoptosis. We also show that miR-338-3p may function as an tumor suppressor by directly targeting SSX2IP. Thus, our results suggest that miR-338-3p is a potential diagnostic marker and therapeutic target of gastric cancer.

## Materials and Methods

### 1. Cell Lines and Culture

The human gastric cancer cell lines SGC-7901, NCI-N87, BGC-823 and AGS were purchased from Shanghai Institutes for Biological Sciences, Chinese Academy of Sciences [23]–[26]. KATO-III and SNU-1 were purchased from the ATCC (American Type Culture Collection) [24], [27], MKN28 and MKN45 were obtained from the Japanese Cancer Research Resources Bank [28], the immortalized gastric epithelial cell line, GES-1, was a gift from Prof. Feng Bi (Huaxi Hospital, Sichuan University, Chengdu, Sichuan Province, PR China) [29], [30]. HEK293T, human embryonic kidney cell line, was preserved in our institute. The gastric cancer cell lines were cultured in RPMI1640 supplemented with 10% FBS(Fetal bovine serum) at 37°C in a humidified atmosphere of 5% CO2. HEK293T cells were cultured in DMEM (Dulbecco’s modified Eagle’s medium) supplemented with the same cultured condition above.

### 2. Ethics Statement

Written informed consent in the study was obtained from all participants. The study protocol was approved by the ethics committee of Ruijin Hospital, School of Medicine, Shanghai Jiao Tong University.

### 3. Tissue Samples

Primary gastric cancer tissues and the matched non-tumor tissues were collected from 66 patients undergoing radical gastrostomy at the Department of Surgery, Ruijin Hospital, Shanghai Jiao Tong University School of Medicine. None of the patients received preoperative treatment. All of the tissue samples were collected with patients’ informed consent and confirmed by the pathological examination, and the histological typing was based on the Lauren’s classification. The TNM classification definitions were according to the International Union against Cancer (2002).

### 4. RNA Isolation and qPCR

Total RNA was isolated from cell lines and tissue samples by *mir*Vana™ miRNA Isolation Kit (Applied Biosystems, Foster City, CA, USA) according to the manufacturer’s instructions. The quality and quantity of the RNA samples were assessed by standard electrophoresis and spectrophotometric methods. The expression level of miR-338-3p in cell lines and tissue samples was measured by qPCR and calculated as described [13]. The expression level of SSX2IP mRNA was measured by qPCR according to the TaqMan® Gene Expression Assays protocol (Applied Biosystems). The GAPDH mRNA level was used for normalization.

### 5. DNA Isolation and Methylation Analysis

Genomic DNA from tissue samples was purified using DNAzol (Invitrogen). Sodium bisulfite conversion was conducted using the Qiagen Epitect Bisulfite Kit (Qiagen) according to the manufacturer. The methylation statues was performed by Methylation specific PCR (MSP). The primers for methylated or unmethylated DNA were designed by the software Methyl Primer Express v1.0 (ABI).The methylation forward primer for the CpG of miR-338-3p : 5′-GGCGGAGTTTATGGTTTTC-3′ and the reverse primer: 5′-AAACCTAACCGATCCTCG-3′, the unmethylation forward primer for the CpG of miR-338-3p: 5′-TGGTGGAGTTTATGGTTTTT-3′ and the reverse primer: 5′-AAACCTAACCAATCCTCACAA-3′.

### 6. Transient Transfection of miRNA Mimics

miRNA-338-3p mimics and negative control mimics were purchased from GenePharma (Shanghai, China).Cells were seeded into cell culture plates 20 h before transfection to ensure 70% cell confluence at the moment of transfection. Transfection of miRNA mimics into cells was carried out using Lipofectamine2000 (Invitrogen) according to the manufacturer’s procedure. The miRNA mimics worked at the final concentration of 100 nM. At 48 h post-transfection, qPCR and Western blot were performed.

### 7. Cell Proliferation Assay

Cell proliferation was assessed by WST (water-soluble tetrazolium salt) assay using a Cell Counting Kit-8 (Dojindo) according to the manufacturer’s instructions. At 24 h post-transfection with miRNA mimics, cells (2×10^3^ cells/well) were seed into 96-well plates. The plates were incubated for 5 days. The number of viable cells was assessed by measurement of the absorbance at 450 nm.

### 8. Soft Agar Colony Formation Assay

GC cells transfected with miRNA mimics were resuspended with 0.3% soft agar in RPMI1640 containing 10% FBS and layered onto 0.6% solidified agar in RPMI1640 containing 10% FBS in 6-well plates (1×10^3^ cells/well). The plates were incubated at 37°C in a humidified atmosphere of 5% CO_2_. Colonies containing at least 50 cells were counted.

### 9. Cell Migration and Invasion Assay

Migration of cells was performed using QCM^TM^24-Well Colorimetric Migration Assay Kit (Millipore) according to the manufacturer’s instructions. For the invasion assay, Cell Invasion Assay Kit (Millipore) was used according to the manufacture’s instructions. Cells (1×10^5^) in 300 µl serum-free medium were added to the upper chambers and cultured for 48 h. Non-migrating or non-invading cells were removed with cottons swabs, Cells that migrated or invaded to the bottom of the membrane were stained with the cell stain buffer provided in the assay kit and counted under microscope and photographed. Three independent experiments were performed for the same conditions.

### 10. Apoptosis Analysis

Cell apoptosis analysis was preformed with Annexin V-FITC Apoptosis Detection Kit I (BD) according to the manufacturer’s instructions. Each assay was performed in triplicate and repeated three times independently. The feature of nuclear shrinkage associated with apoptosis was showed by Hoechst 33342 (Beyotime ) according to the protocol.The morphology was visualized by a fluorescent microscope that was excited at wavelength 350 nm and measured at 460 nm.

### 11. Construction of Plasmids and Luciferase Activity Assay

The fragment of wild-type (wt) 3′UTR of SSX2IP or sites mutant (mut) 3′UTR of SSX2IP containing the putative miR-338-3p binding sites were synthesized. The fragments were cloned into the pMIR-Report luciferase vector (Ambion) containing *Firefly* and named pMIR/SSX2IP-3′UTR^wt^ and pMIR/SSX2IP-3′UTR^mut^.Cells were seed into the 24-well plates 24 h before transfection. 500 ng of pMIR/SSX2IP-3′UTR^wt^ or pMIR/SSX2IP-3′UTR^mut^ were transfected into each well, together with 20 ng of pRL-TK vector (Promega) containing *Renilla* luciferase and 60 pmol of the miR-338-3p or control mimics. Cells were harvested after 48 h transfection. *Firefly* and *Renilla* luciferase activities were measured by using Dual-luciferase reporter assay (Promega) according to the manufacturer’s instructions.

### 12. Stable Transfection of miR-338-3p Expression Vector

pSilencer-miR-338-3p vector and pSilencer-miR-control vector was transfected into SGC-7901 cells respectively using Lipofectamine2000 (Invitrogen), and selected with 100 µg/ml hygromycin B (Invitrogen) for 4 weeks. Stably transfected cell clones were picked and maintained in half of the selection concentration.

### 13. Tumor Xenograft Model

SGC-7901 cells (2×10^6^ cells/mouse) stably transfected with pSilencer-miR-338-3p or pSilencer-control vector were subcutaneously injected into 4-week-old male nude mice (Institute of Zoology, Chinese Academy of Sciences, Shanghai, China).The mice were checked weekly, the tumor nodules were measured with a caliper. Mice were euthanized 4 weeks after injection, and the tumors were removed and weighed. Tumor volume was evaluated using the following formula: Volume = (width+length)/2×width×length× 0.5236.Tumor growth curves were calculated.

### 14. Immunohistochemistry

Detection of SSX2IP was performed on paraffin sections with the anti-SSX2IP antibody (Abcam, dilution 1∶1000). The immune complex was visualized with the Dako REAL^TM^EnVision™ Detection System, Peroxidase/DAB, Rabbit/Mouse (Dako), according to the manufacturer’s procedure. The nuclei were counterstained with hematoxylin.

### 15. Statistical Analysis

The relationship between the miR-338-3p expression level and clinicopathologic parameters was analyzed by the Person *X* ^2^ test. The differences between groups were analyzed using Student *t* test. All statistical analyses were performed using the SPSS 13.0 software (SPSS Inc, Chicago, IL, USA), and *P*<0.05 was considered significant.

## Results

### 1. The Expression of miR-338-3p is Down-regulated in GC Cell Lines and Tissues

**Figure 1:**
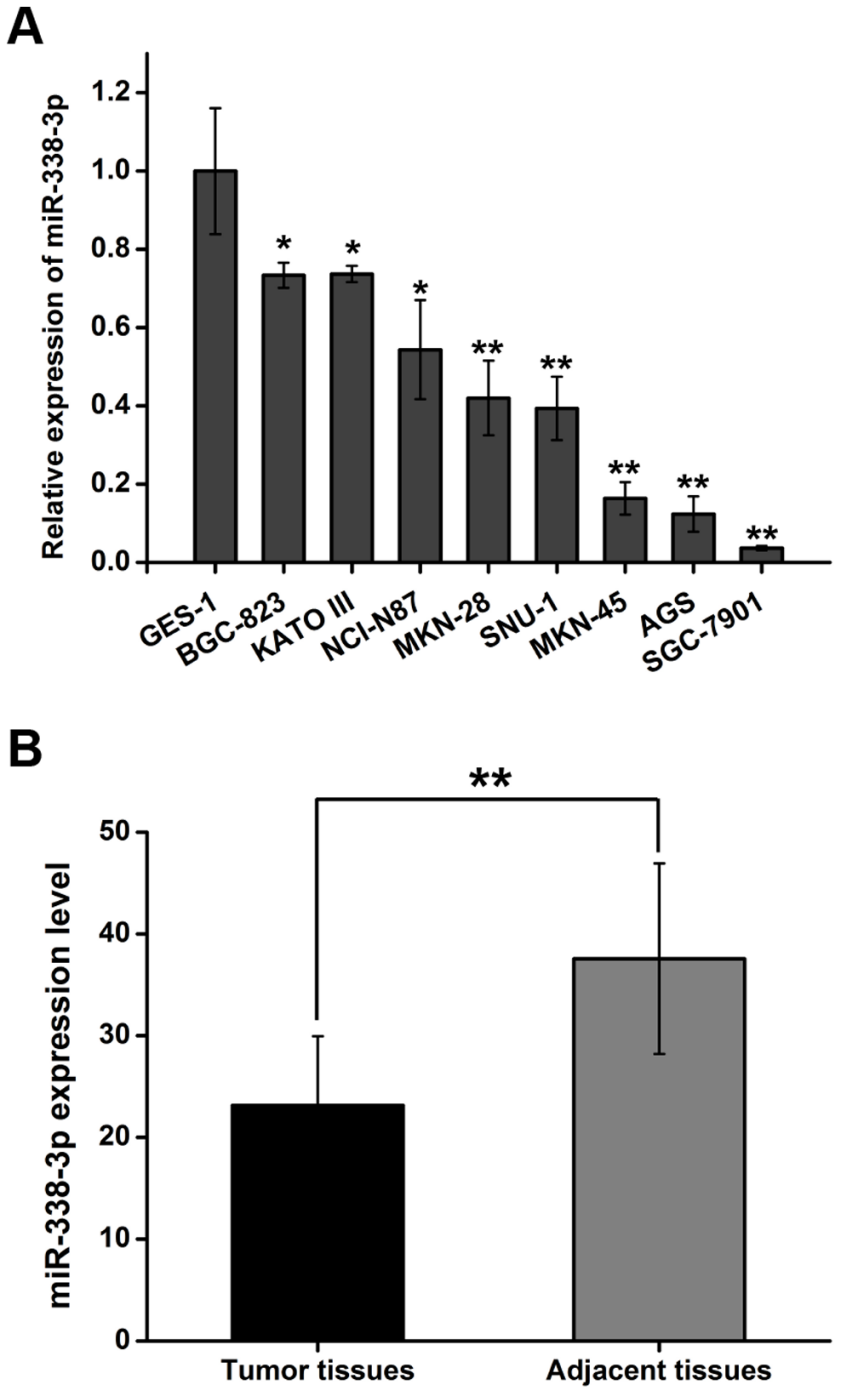
Downregulation of miR-338-3p expression in gastric cancer cell lines and GC tissues compared with the corresponding controls. (A) Relative expression of miR-338-3p in gastric cell lines and the immortalized normal gastric mucosal epithelial cell line (GES-1) (*P<0.05, **P<0.01) were detected by qPCR. (B) qPCR for expression of miR-338-3p was carried out using 66 cases gastric cancer tissues and matched adjacent tissues. The mean and standard deviation were shown (**P<0.01).

To explore the role of miR-338-3p in GC, we first compared the expression level of miR-338-3p in eight gastric cancer cell lines with an immortalized normal gastric mucosal epithelial cell line (GES-1). As shown in Fig. 1A, miR-338-3p was significantly down-regulated in GC cell lines compared with GES-1. To confirm this result, we also compared the expressions of miR-338-3p in GC tissues and adjacent non-tumor tissues. 66 GC paired samples were included in this study. The results show that the average expression level of miR-338-3p was significantly down-regulated in tumor tissues compared to the adjacent non-tumor tissues (Fig. 1B). Furthermore, we elucidated the correlation between miR-338-3p expression and clinicopathologic factors. As shown in Table 1. 59.1%(39/66) of GC samples showed down-regulation of miR-338-3p below two-fold cut-off (relative expression ratio <0.5), this situation was considered to be significantly down-regulated. Based on this cut-off, the 66 clinical cases were divided into two groups: miR-338-3p lower expression group (n = 39) and miR-338-3p higher expression (n = 27). The lower expression group showed inclination towards larger tumor size, more lymph node metastasis, deeper local invasion and more advanced TNM stage, but no significant correlation with age, gender or histological type (Table 1).

**Table 1:** Relationship between miR-338-3p expression level and clinicopathologic variables in 66 GC patients.

Clinicopathologic parameters	miR-338-3p expression		*P*
	low (n = 37)	high (n = 29)	
*Age*			
≤60	16	17	0.215
>60	21	12	
*Gender*			
Male	22	17	0.945
Female	15	12	
*Diameter (cm)*			
≤5	16	20	0.037
>5	21	9	
*Location*			
Distal third	17	10	0.347
Middle third, proximal third	20	19	
*Histologic type*			
Intestinal	19	20	0.149
Diffuse	18	9	
*Local invasion*			
T1,T2	8	15	0.011
T3,T4	29	14	
*Lymph node metastasis*			
No	8	13	0.045
Yes	29	16	
*TNM*			
I,II	6	18	<0.001
III,IV	31	11	

### 2. miR-338-3p is Epigenetically Silenced in Gastric Cancer

**Figure 2:**
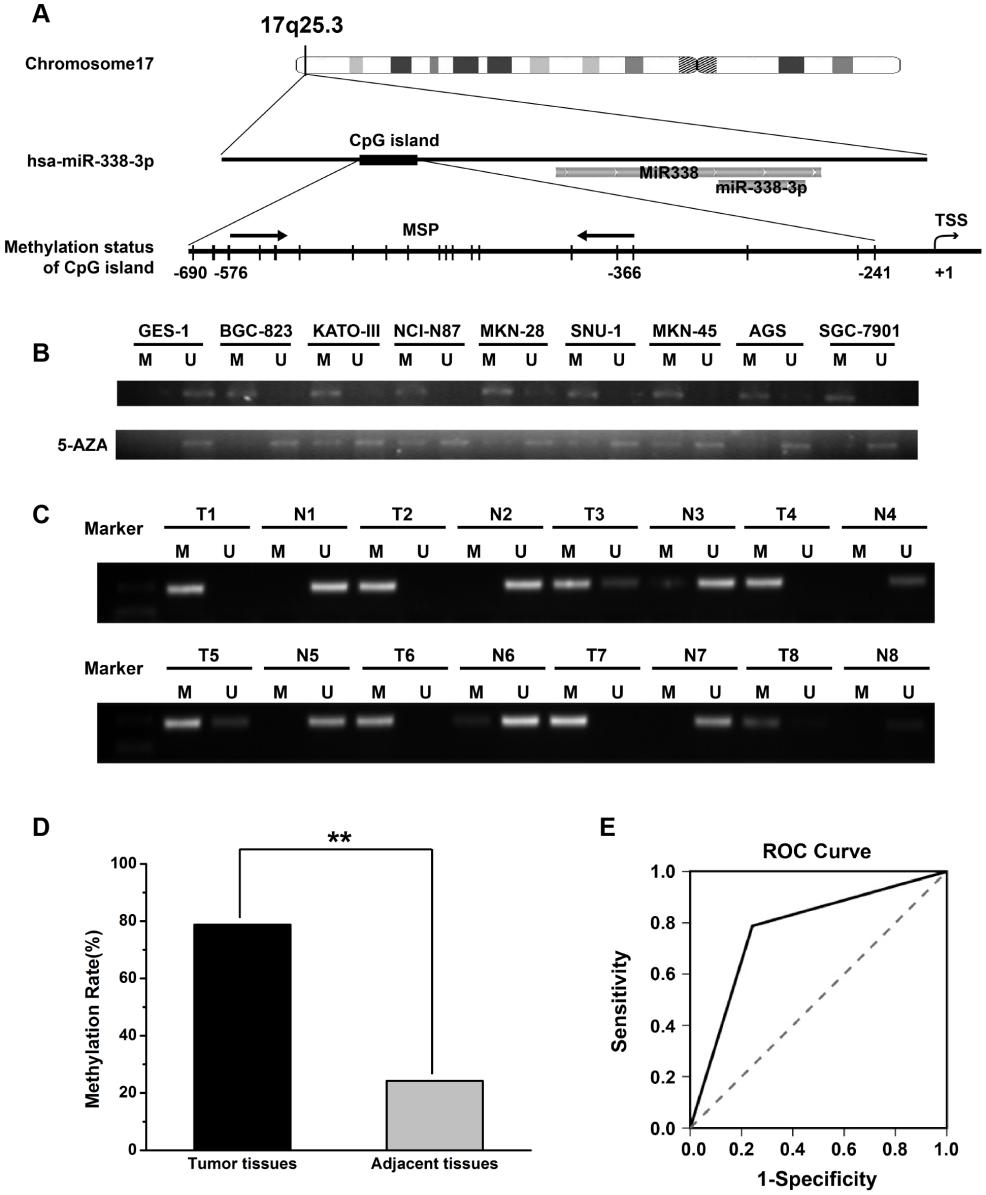
Methylation status of miR-338-3p CpG island. (A) Schematic locations of CpG sites within the CpG islands that surround the miR-338-3p promoter region. (B) MSP analysis for miR-338-3p methylation in GC cell lines. M: MSP of methylation-specific primers; U: MSP of non-methylation-specific primers (C) Representative results of MSP analysis of miR-338-3p in gastric cancer tissues (T) and adjacent normal tissues (N). (D) Methylation levels of miR-338-3p CpG island in GC tumor tissues and matched adjacent non-tumor tissues in 66 patients (78.79% and 24.24%, **P<0.01). (E) The receiver operating characteristics curve of methylation status of miR-338-3p. The AUC was 0.773±0.042 (*P*<0.001), 95% confidence interval was (0.690, 0.856).

As epigenetic silencing is a common means for down-regulation of miRNA expression [31], [32], we decided to analyze the CpG island distribution in the promoter region of miR-338-3p by Methyl Primer Express v1.0 software. The results showed that CpG islands exist in the section (–690 to –241) upstream of the transcription start site (TSS). We examined the methylation status of CpG islands in the promoter region by methylation-specific PCR (MSP), covering the region of –366 to –576 (Fig. 2A). By using MSP, we found that the miR-338-3p CpG island was extensively methylated in the GC cell lines compared to in the gastric mucosal epithelial cell line (GES-1). As expected, treatment with 5-AZA induced significant demethylation (Fig. 2B). The representative results of MSP analysis of miR-338-3p in GC tumor tissues and matched adjacent non-tumor tissues were shown in Fig. 2C. The methylation rates of miR-338-3p CpG islands in tumor tissues and adjacent non-tumor tissues from 66 GC patients were 78.79% and 24.24%, respectively (**P<0.01,Fig. 2D). When the Receiver Operating Characteristics curve and the Area Under Curve (AUC) for methylated status of miR-338-3p in GC were calculated, the AUC was 0.773±0.042, significantly higher than that of the null hypothesis (*P<*0.001), and the 95% confidence interval was 0.690 to 0.856 (Fig. 2E). Thus, the methylation status of miR-338-3p CpG island could serve as a potential molecular marker for GC.

### 3. miR-338-3p Inhibits Gastric Cancer Cells Proliferation

Since miR-338-3p is significantly down-regulated in GC, as shown above, it may function as a tumor suppressor. Therefore, we next determined whether overexpression of miR-338-3p in gastric cancer cells could affect cell growth. Synthetic miR-338-3p mimics or negative control mimics were transfected into SGC-7901 cells in which the expression level of the miR-338-3p is the lowest, followed by WST assay to monitor cell growth.

**Figure 3:**
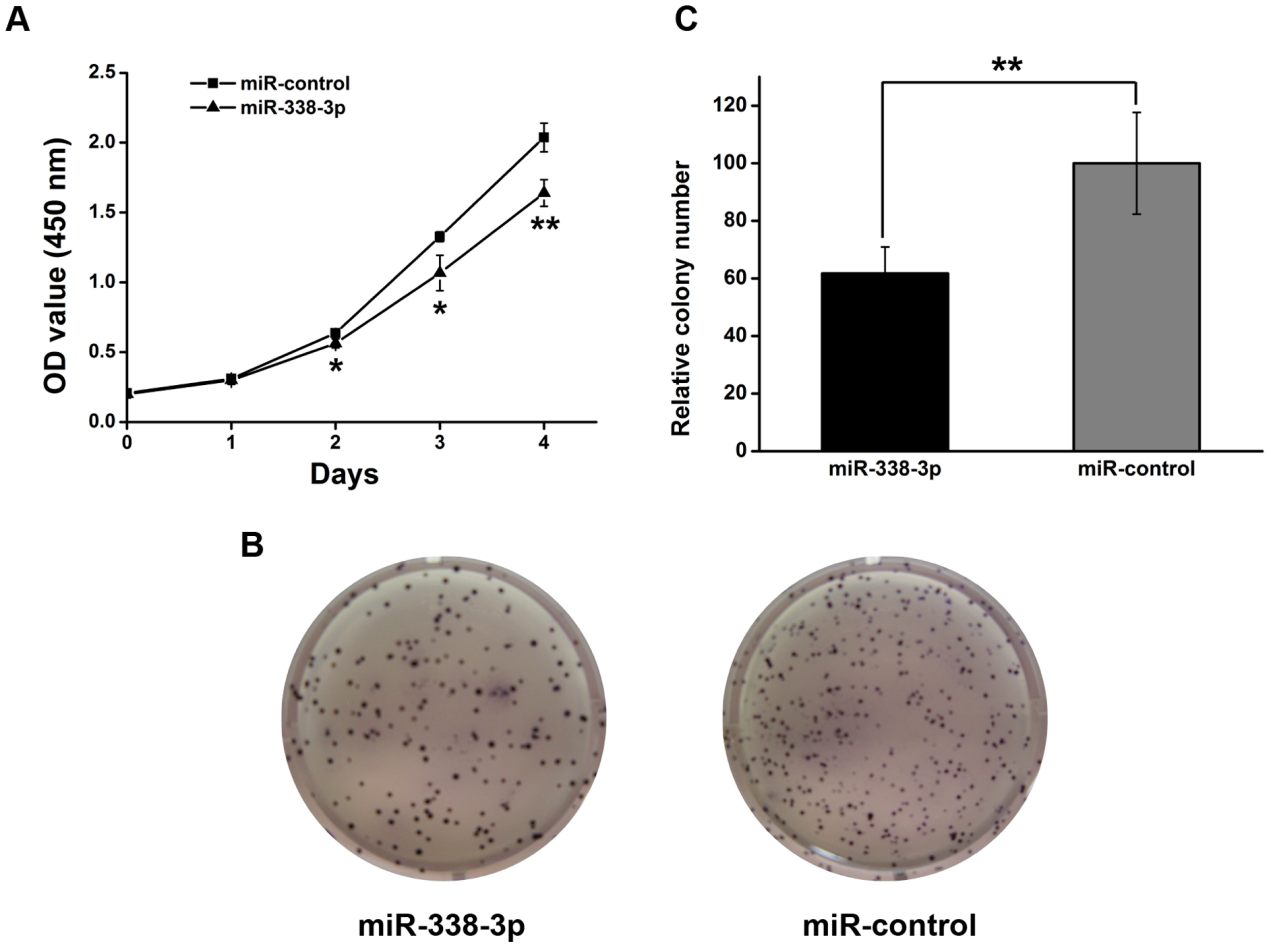
Overexpression of miR-338-3p suppresses the proliferation of SGC-7901 cells. (A) SGC-7901 cells proliferation were performed by the WST assay. SGC-7901 cells were transfected with miR-338-3p mimics or control mimics at a final concentration of 100 nM.The WST assay was performed every 24 h for 4 days. The results are means of three independent experiments ± S.D.(*P<0.05, **P<0.01). (B–C) The effect of miR-338-3p on SGC-7901 cells proliferation was evaluated by colony formation assay. SGC-7901 cells transfected with 100 nM miR-338-3p mimics or control mimics were seeded onto 6 well plates. The number of colonies was counted on the 14^th^ day after seeding. (B) Representative photographs of colonies. (C) Colonies were counted. The results were means of three independent experiments ± S.D. (**P<0.01).

The ectopic expression of miR-338-3p in SGC-7901 cells was confirmed by qPCR and SGC-7901 cells transfected with miR-338-3p mimics grew significantly slower than the control group (Fig. 3A)(*P<0.05, **P<0.01). To further characterize the effect of miR-338-3p on cell growth, soft-ager colony formation assay was performed. We found that the number of colonies from SGC-7901 cells transfected with miR-338-3p mimics was significantly fewer than that of the control group (Fig. 3B–C)(**P<0.01). Consistently, inhibition of miR-338 induced the proliferation of SGC-7901 (Fig. S2 in File S1, *P<0.05). Similar results were observed in the control GES-1 cells (Fig. S1 A–B in File S1, *P<0.05). In summary, these results suggested that miR-338-3p suppresses the growth rate of both GC cells and gastric epithelial cells *in* *vitro.*

### 4. miR-338-3p Inhibits Migration and Invasion of GC Cells *in vitro*

**Figure 4:**
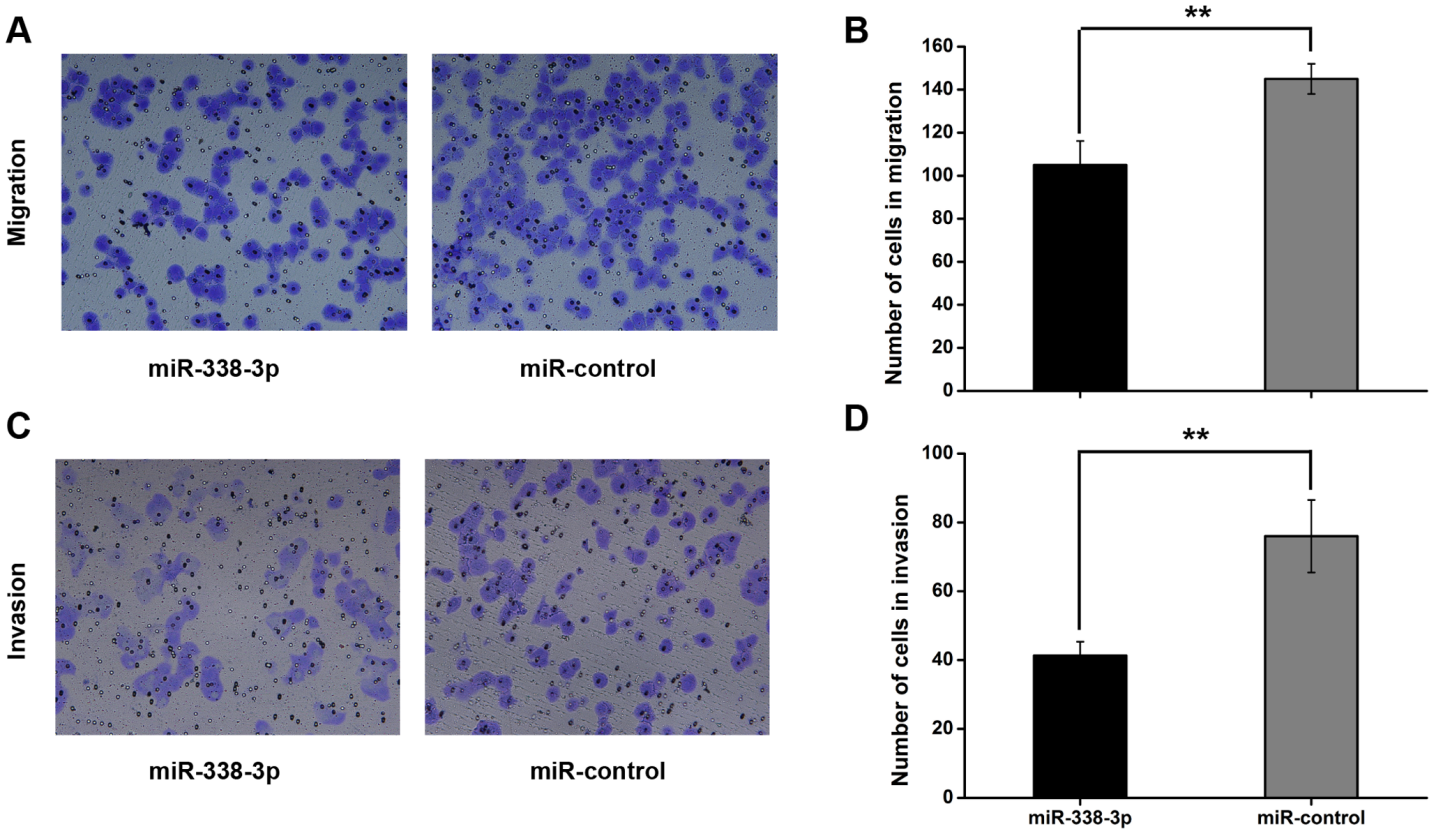
miR-338-3p inhibits migration and invasion of SGC-7901 cells *in vitro.* (A) Representative photographs of migratory cells on the membrane (magnification 100×).(B) Average migratory cell number of three independent experiments ± S.D. (**P<0.01). (C) Representative photographs of invasive cells on the membrane (magnification 100×). (D) Average invasive cell number of three independent experiments ± S.D. (**P<0.01).

Based on the idea that miR-338-3p serves as a tumor suppressor of GC, we further explored its effects on cell migration and invasion, a critical determinate of malignant progression and metastasis. As shown in Fig. 4, the number of migratory SGC-7901 cells transfected with miR-338-3p mimics was significantly less than the control group (105.00±11.16 vs 145.00±7.00, **P<0.01. Fig. 4 A–B), and number of invasive SGC-7901 cells transfected with miR-338-3p was significantly less compared to the control group (41.33±4.04 vs 76±10.54, **P<0.01. Fig. 4 C–D). Consistently, inhibition of miR-338-3p induced migration and invasion of SGC-7901 cells (Fig. S3 in File S1, *P<0.05). By contrast, miR-338-3p had no influence on migration of GES-1 (Fig. S1 E in File S1), likely because GES-1 cells lack the migration and invasive nature of cancer cells. These results displayed a role of miR-338-3p in inhibiting both migration and invasion of GC cells.

### 5. miR-338-3p can Induce Gastric Cancer Cells Apoptosis

**Figure 5:**
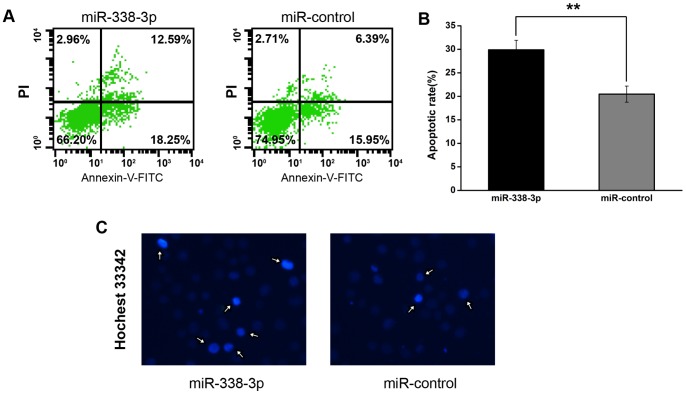
The effect of miR-338-3p on apoptosis of GC SGC-7901 cells. (A) Representative histograms depicting apoptosis of SGC-7901 cells transiently transfected with miR-338-3p mimics or control mimics. Cells were stained with PI and Annexin V-FITC at 48 h post-transfection. (B) The percentage of apoptotic cells of three independent experiments ± S.D. are shown (**P<0.01). (C) Representative histograms depicting nuclear morphology of SGC-7901 cells transiently transfected with miR-338-3p mimics or control mimics cells with Hoechst33342 (magnification 200×).

To elucidate the mechanism of miR-338-3p on GC cell growth inhibitory effects, we tested apoptosis analysis by flow cytometry. The results indicated that the apoptotic rate was significantly increased in miR-338-3p mimics transfected group compared to the control mimics transfected group (**P<0.01)(Fig. 5A–B). We further examined the morphologic features of apoptosis including condensed chromatin and nuclear fragmentation by Hoechst 33342 staining, and reaffirmed that the apoptotic rate was increased in miR-338-3p mimics transfected cells (Fig. 5C). Consistently, inhibition of miR-338 inhibited apoptosis of SGC-7901 cells (Fig. S4 in File S1, *P<0.05). Similar results were observed in the control GES-1 cells (Fig. S1 C–D in File S1, **P<0.01). These results indicated that overexpression of miR-338-3p induces apoptosis in GC cells, which could contribute to the growth inhibitory properties of miR-338-3p.

### 6. miR-338-3p Inhibits Tumorigenicity and Increases Apoptosis *in vivo*

**Figure 6:**
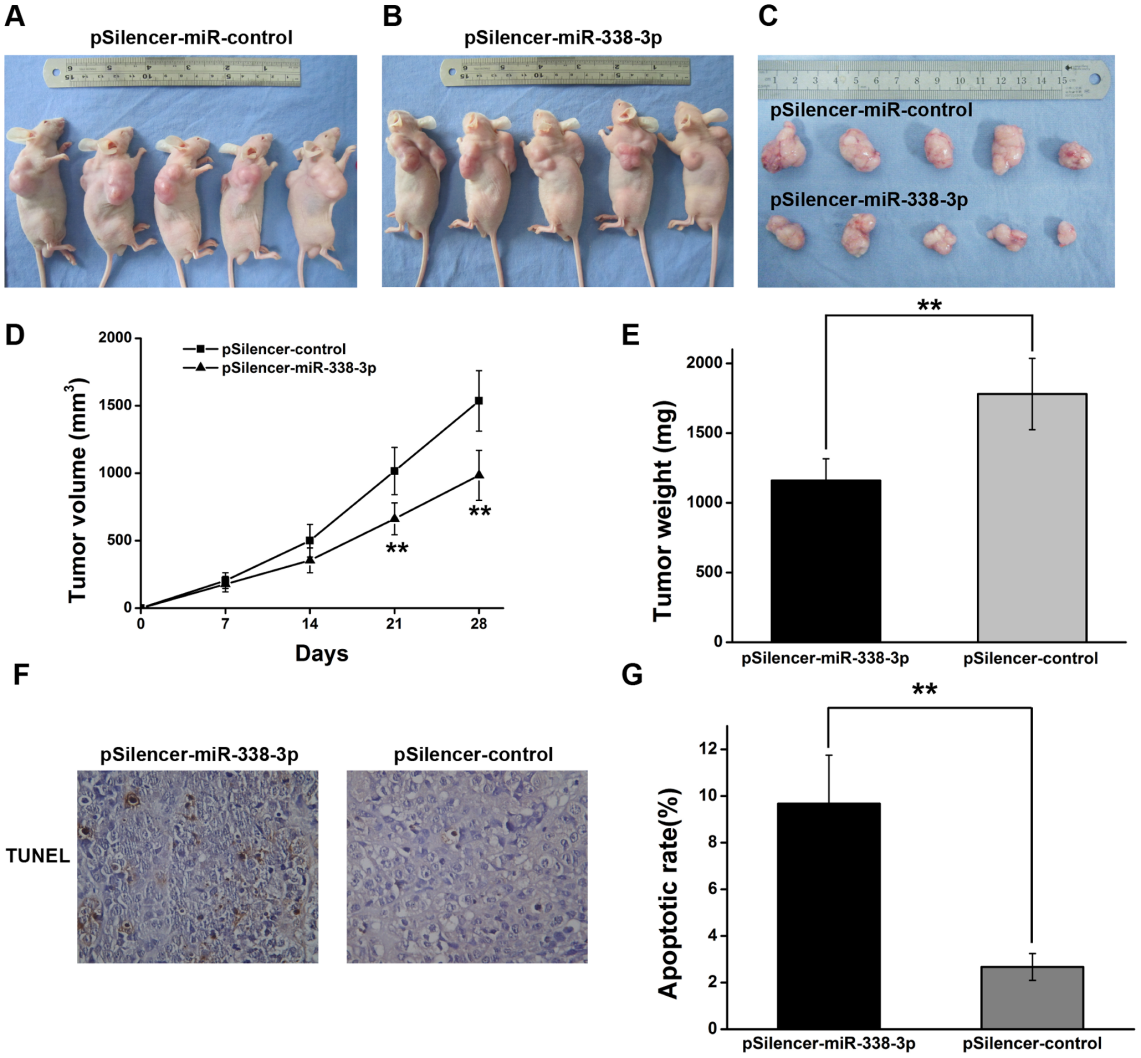
miR-338-3p inhibits tumorigenicity and increases apoptosis in* vivo.* (A–C) Photographs of tumors derived for pSilencer-miR-338-3p or pSilencer-control vector stably transfected SGC-7901 cells in nude mice. (D) Growth kinetics of tumors in nude mice. Tumor diameters were measured every 7 days (**P<0.01). (E) Average weight of tumors in nude mice. Means ± S.D. were shown (**P<0.01). (F) Representative photographs of TUNEL assay of tumor specimens from nude mice (magnification, 200×). (G) The percentage of apoptotic cells was counted. Means ± S.D.(**P<0.01).

We next tested whether ectopic expression of miR-338-3p could influence the growth and apoptosis of gastric tumor *in vivo.* SGC-7901 cells were transfected with the miR-338-3p expression vector (p*Silencer*-miR-338-3p) or the control vector (p*Silencer*-miR-control). The stable clones with either p*Silencer*-miR-338-3p or p*Silencer*-miR-control were selected and injected subcutaneously into nude mice, and the tumor formation was monitored. SGC-7901 cells transfected with miR-control showed progressive growth but were inhibited when ectopic expression of miR-338-3p. After 28 days, the nude mice were euthanized, and the tumor weights and volume were measured (Fig. 6 A–E). The average weight of tumors resulting from pSil*encer*-miR-338-3p cells group was significantly less than tumors from p*Silencer*-miR-control cells group (1200±193.26 mg vs 1800±229.52 mg, **P<0.01).

To examine whether apoptosis contributes to this observed *in vivo* tumor growth inhibition upon miR-338-3p over-expression, TUNEL assay was performed in the tumor tissues. The results showed that tumor tissues from p*Silencer*-miR-338-3p contained significantly more positive staining cells compared with control group (**P<0.01, Fig. 6F–G). Thus, our results indicated that miR-338-3p inhibits gastric tumorigenesis *in vivo*, at least partly by inducing apoptosis in situ.

### 7. miR-338-3p Targets the 3′-UTR of the Oncogene *SSX2IP*

As miRNAs often regulate the expression of target genes, we searched miR-338-3p’s putative targets using online search tools (e.g. TargetScan, Microrna.org and PicTar). The genes predicted by all the used programs were considered candidate targets of miR-338-3p. Among all the hits, SSX2IP caused our attention, previous studies indicated that SSX2IP is an acute myeloid leukemia-associated antigen and a potential immunotherapy target for leukemia [33]–[35] and our previous work showed that SSX2IP promotes the invasion and migration of hepatocellular carcinoma cells and contributes to the chemotherapy resistance [36].

**Figure 7:**
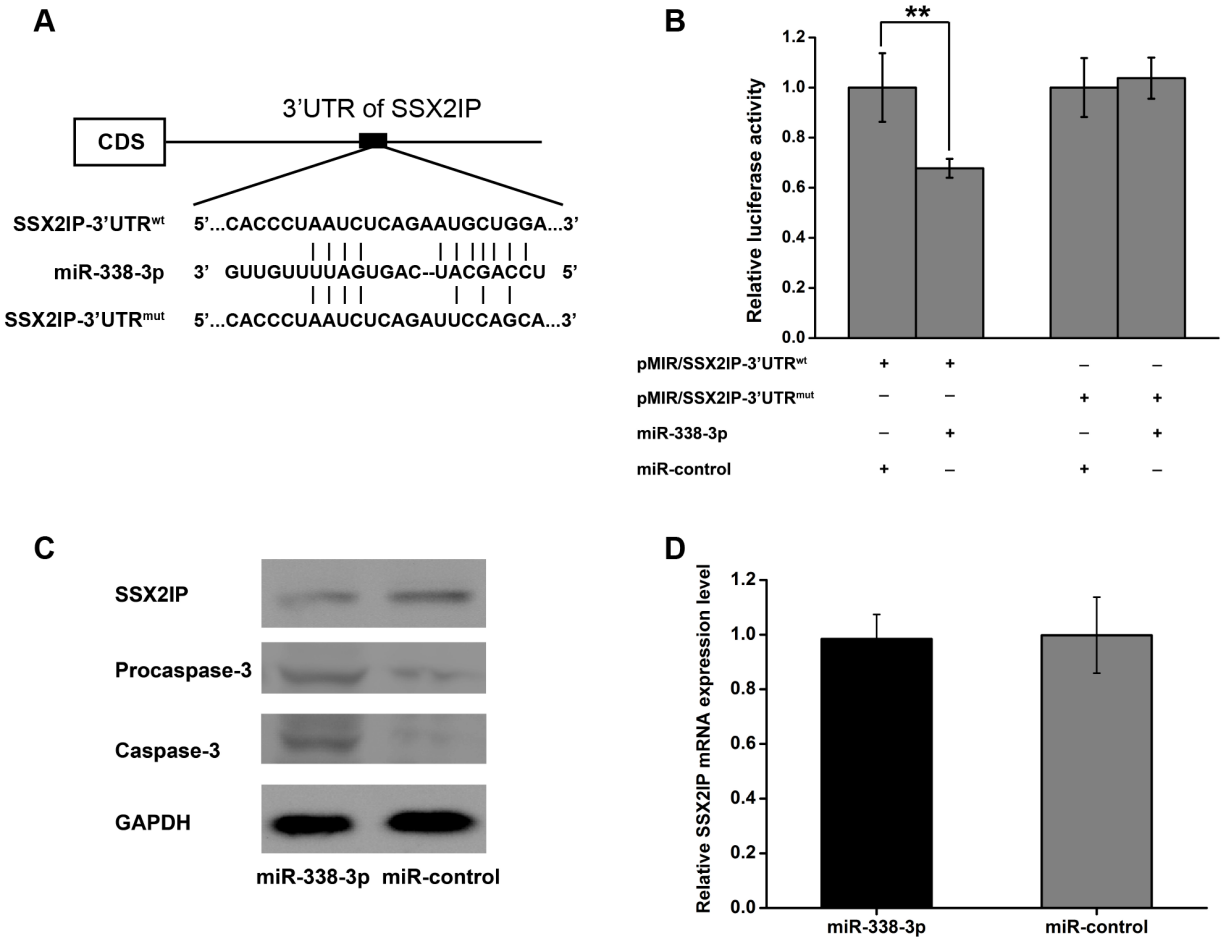
miR-338-3p targets 3′UTR of *SSX2IP* and induces Caspase-3 expression. (A) Schematic graph of the putative binding sites of miR-338-3p targeted the 3′UTR of SSX2IP. (B) miR-338-3p mimics downregulated luciferase activities controlled by wild-type 3′UTR of SSX2IP(**P<0.01), but did not affect luciferase activity controlled by mutant 3′UTR of SSX2IP. The results are means of three independent experiments ± S.D. (C) SSX2IP, Caspase-3 and Pro-casepase-3 were detected by Western blot at 48 h post-transfection with miR-338-3p and control mimics. (D) SSX2IP mRNA in SGC-7901 cells analyzed by qPCR at 48 h post-transfection with miR-338-3p and control mimics.

One potential miR-338-3p binding site was predicted within the 3′UTR of SSX2IP (Fig. 7A). To validate this interaction, we put a wild-type or mutant 3′UTR fragment of SSX2IP into the pMIR-REPORT luciferase vector. The resultant constructs were co-transfected with miR-338-3p mimics or control mimics into HEK293T cells, followed by luciferase reporter assay. The relative luciferase activity of pMIR/SSX2IP-3′UTR^wt^, but not pMIR/SSX2IP-3′UTR^mut^, was significantly suppressed by miR-338-3p (**P<0.01) compared with that of the control mimics (Fig. 7B), indicating that miR-338-3p directly binds to the 3′UTR of SSX2IP to function as a suppressor.

We next examined the influence of miR-338-3p on the expression of SSX2IP, by measuring the mRNA and protein levels of SSX2IP after transfecting SGC-7901 cells with miR-338-3p or control mimics. As shown in Fig. 7C–D, miR-338-3p had no effect on the *SSX2IP* mRNA level as measured by qPCR, but caused significant reduction of the SSX2IP protein level compared with control-transfected. These results suggest that miR-338-3p suppresses the expression of SSX2IP at the post-transcriptional level, likely through binding to the 3′UTR of SSX2IP. Notably, we also found that capase-3 and procasepase-3, two important apoptosis genes, were decreased accordingly (Fig. 7C).

### 8. The Expression of miR-338-3p is Inversely Correlated with the Expression Level of SSX2IP Protein in Gastric Cancer

miR-338-3p is down regulated in gastric cancer and SSX2IP is supported to a target gene of miR-338-3p, it promoted us to speculate that SSX2IP might be overexpression in gastric cancer tissues and relative to matched non-tumor tissues.

**Figure 8:**
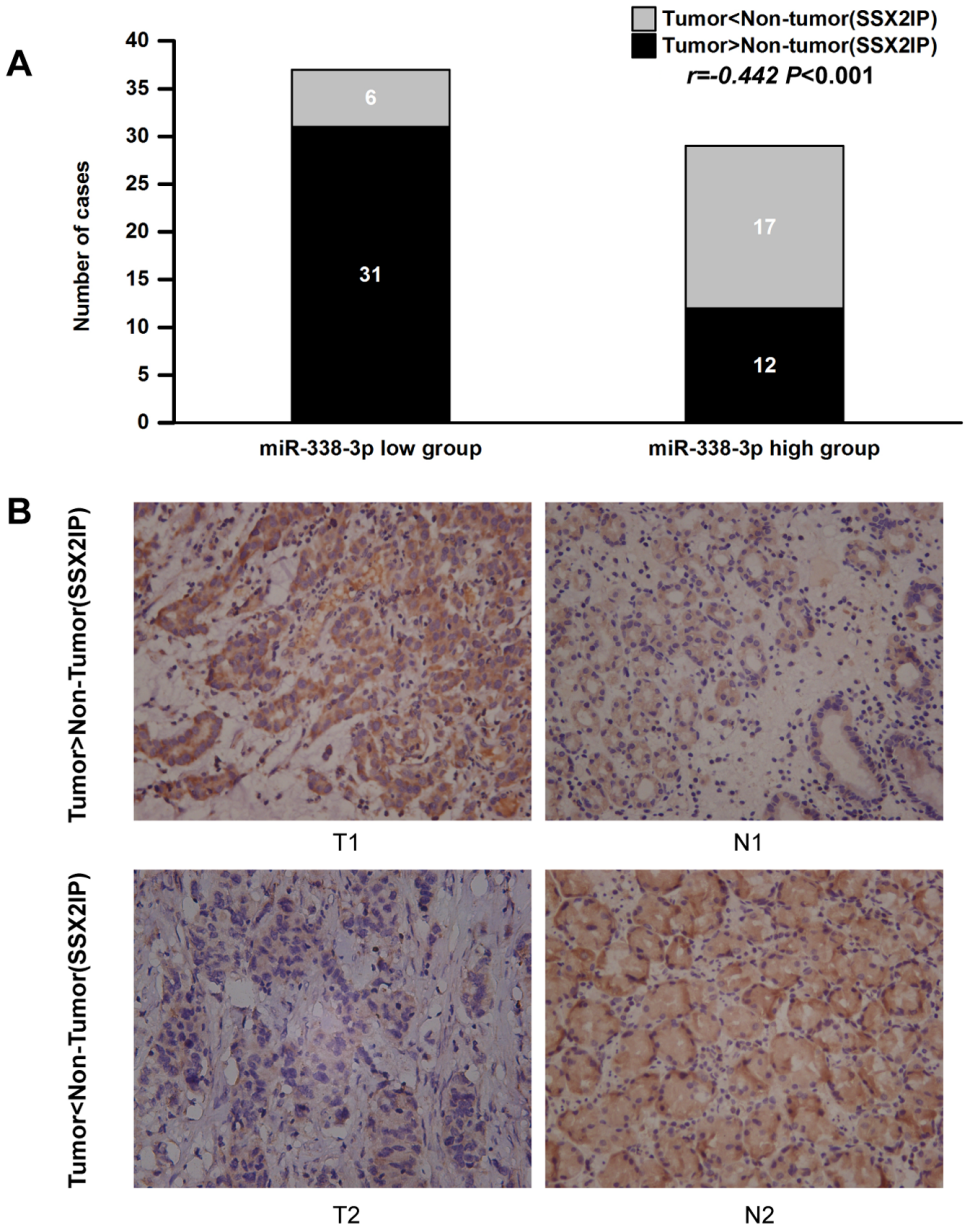
miR-338-3p expression correlates inversely with SSX2IP protein expression in gastric cancer. (A) Immunohistochemical analysis showed that increased expression of SSX2IP was more frequently observed in gastric cancer tissues in miR-338-3p low-expression group and vice versa. Tumor>Non-tumor (SSX2IP) means overexpression of SSX2IP in GC tumor tissue compared with matched non-tumor tissue, and vice versa. (B) Representative photographs of immunohistochemical analysis of SSX2IP in paired GC tumor/non-tumor tissues (magnification, 200×).

Immunohistochemical analysis of SSX2IP antigen revealed the situation of staining for SSX2IP protein in tumor tissues and matched non-tumor tissues in 66 gastric cancer samples.65% (43/66) of gastric cancer tissues displayed elevated immuostaining for SSX2IP protein compared with matched non-tumor tissues. In particular, 84% (31/37) of gastric cancer tissues displayed elevated immunostaining in GC samples of the miR-338-3p low-expression group, while the data is 41% (12/29) in the miR-338-3p high-expression group. Thus, the analysis of the expression of miR-338-3p and SSX2IP in gastric cancer tissues confirmed the existence of inverse correlation between the expression of miR-338-3p and its target gene SSX2IP (Person Chi-square test: *P*<0.001, Spearman Correlation: r = -0.442, *P*<0.001, Fig. 8).

### 9. Ectopic Expression of SSX2IP Rescues the Phenotypes Caused by Over-producing miR-338-3p in GC Cells

**Figure 9:**
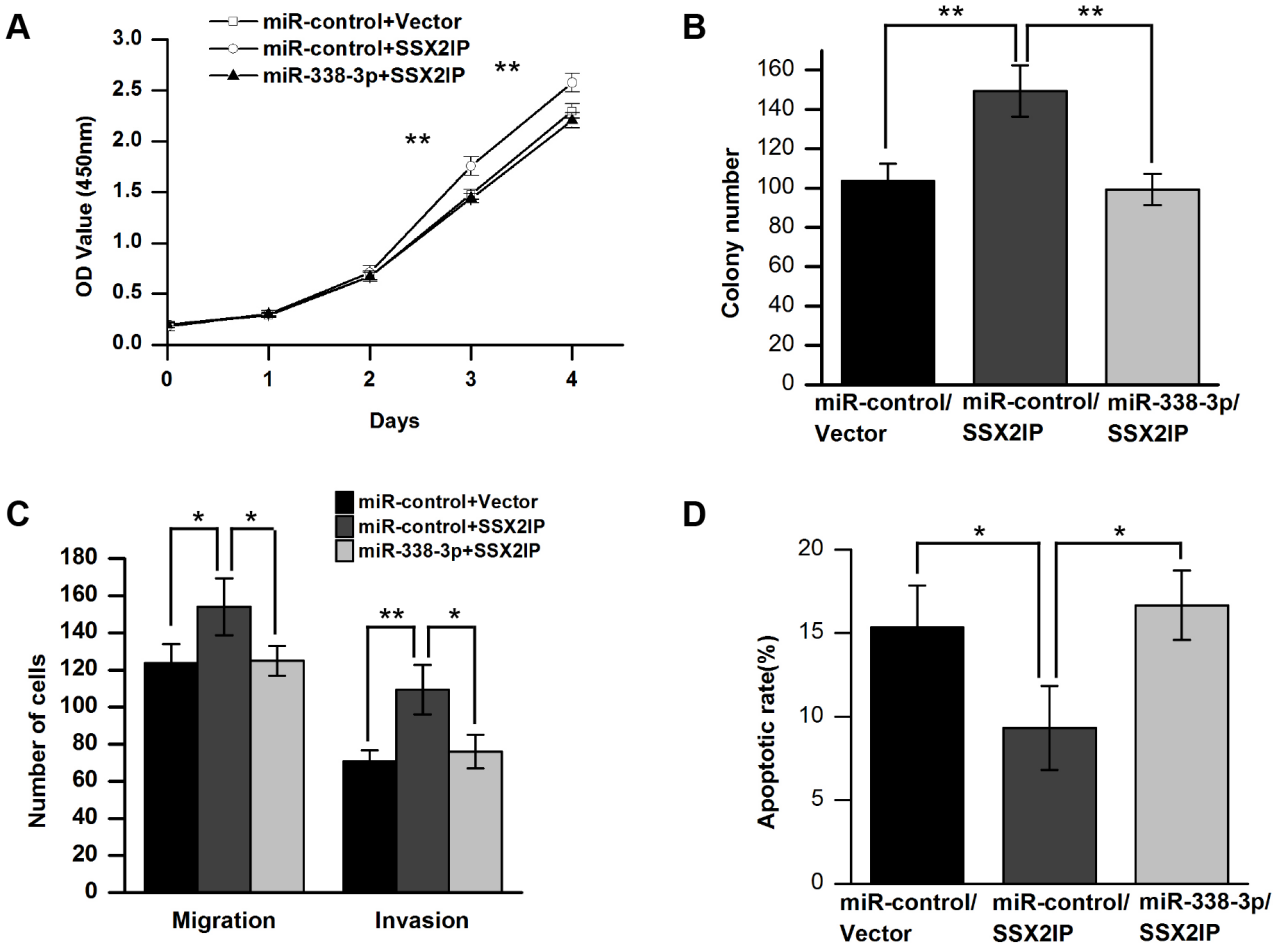
SSX2IP can reverse the biological phenomena induced by miR-338-3p. (A) SGC-7901 cells proliferation was performed by the WST assay. The WST assay was performed every 24 h for 4 days. The results are means of three independent experiments ± S.D.(**P<0.01). (B) The effect of SSX2IP on SGC-7901 cells was evaluated by colony formation assay. SGC-7901 cells were seeded onto 6 well plates. The number of colonies was counted on the 14^th^ day after seeding. The results were means of three independent experiments ± S.D. (*P<0.05, **P<0.01). (C) Average migratory and invasive cells numbers of three independent experiments ± S.D. (**P<0.01). (D) The effect of SSX2IP on apoptosis of GC SGC-7901 cells was performed by Annexin V Kit. The percentage of apoptotic cells of three independent experiments ± S.D. are shown (*P<0.05).

If SSX2IP is a main target suppressed by miR-338-3p in GC, ectopic expression of SSX2IP should rescue the phenotypes caused by miR-338-3p over production in GC cells. To test this idea, and SSX2IP expression vector or empty vector was individually transfected into SGC-7901, followed by cytological assays to measure cell proliferation, migration, invasion and apoptosis. As expected, overexpression of SSX2IP partially or fully reverses the inhibitory effect of miR-338-3p in GC cells on each aspect we examined (Fig. 9 A–D). On the other hand, SSX2IP has no influence on the expression of miR-338-3p (Fig. S5 in File S1). Thus, we concluded that the cancer suppressor function of miR-338-3p in GC is at least partially through suppressing its target gene SSX2IP.

## Discussion

In this study, we found that the low-expression of miR-338-3p was clinically associated with larger tumor size, more lymph node metastasis, deeper local invasion and advanced TNM stage. At the cytological level, up-regulation of miR-338-3p inhibited proliferation, colony formation, invasion and migration of gastric cancer cells. Moreover, over-expressing miR-338-3p in gastric cancer cells significantly reduced tumorigenesis in nude mice *in vivo*. Taken together, our results strongly suggest a tumor suppressor role of miR-338-3p for gastric cancer.

MiRNAs have been recently recognized as crucial tumor-associated regulatory factors. Although the regulatory mechanism of miRNAs expression in tumorigenesis is still unclear, epigenetic regulation has been suggested to play an important role [31], [32]. DNA methylation occurs mainly in the CpG island commonly located in the promoter region of the gene, approximately 70% of the promoter regions in human genome is rich in CpG island [37]. The reduced expressions of miR-34b, miR-129 and miR-10b are all caused by hypermethylation in promoters [38], [39]. Consistent with this notion, the methylation level of CpG island of miR-338-3p in gastric cancer tissues was indeed found higher than that of the matched adjacent non-tumor tissues, suggesting that miR-338-3p is epigenetically silenced in GC. The cause of this epigenetic silence of miR-338-3p in gastric cancer remains elusive, and we speculate that factors like CagA, which has been previously reported to epigenetically affect the expression of microRNAs in gastric cancer [40], may participate in this regulation. Importantly, the hyper-methylation status of miR-338-3p promoter is a potential biomarker for gastric cancer diagnosis.

Since miRNAs function through regulating the expression of their downstream genes, the key question concerning how miR-338-3p contributes to GC is which gene(s) it regulates.

Here we identified SSX2IP as an important novel target of miR-338-3p, and provided both cytological and histological evidence suggesting the tumor-promoting role of SSX2IP in GC. Many studies indicated that SSX2IP is an acute myeloid leukemia-associated antigen and a potential immunotherapy target for leukemia [33]–[35]. Importantly, SSX2IP was shown in our previous studies to promote the motility, invasion and migration and chemotherapy resistance of hepatoma cells, indicating its important role in the development and metastasis. Its homologous gene in rodents is *ADIP* (afadin DIL domain-interacting protein), the amino acid sequence of ADIP protein in mouse and rat showed 88% and 87% identity with SSX2IP protein, respectively. ADIP localized in cell-cell adherens junctions, and it can promote cell mobility by activating Rac1 through Vav2 [41], [42]. It is speculated that SSX2IP could promote cell motility, invasion and metastasis through activating Rac1.

Interestingly, the expression of Rac1 in gastric cancer tissues was higher than in the matched adjacent non-tumor tissues, its expression level was significantly associated with TNM stage. In 7 gastric cancer cell lines, the expressions of Rac1 were higher than in gastric mucosal epithelial cell line (GES-1) [43], which was coincided with the phenomenon we found with miR-338-3p in gastric cancer cells and tissues. These results suggest that miR-338-3p, as a inhibitory factor of gastric cancer, may reduce the expression of target gene SSX2IP to suppress Rac1 activation. The involvement of SSX2IP in multiple cancers reinforces the biological and physiological significance of the miR-338-3p/SSX2IP cascade in cancer development, and has prompted us to further investigate the status of miR-338-3p/SSX2IP in other types of cancers. Identifying other potential targets of miR-338-3p is also a work in progress.

In summary, this study first described miR-338-3p as a tumor suppressor, which is frequently silenced through epigenetic hypermethylation at the promoter region in gastric cancers. Functionally, miR-338-3p inhibits the metastatic features of GC cells such as proliferation, migration and invasion *in vitro*, and reduces the tumorigenicity of GC cells by inducing apoptosis *in vivo*. Moreover, we identified SSX2IP as a crucial target gene of miR-338-3p in GC development. Our results raise the possibility of using miR-338-3p as a early biomarker and therapy target of gastric cancer, and call for a thorough understanding of its regulatory and functions of its downstream target genes has a very important significance.

## Supporting Information

File S1 **Supporting information figures. Figure S1. miR-338-3p inhibits GES-1 proliferation and induces apoptosis, but has no influence on migration.** (A) GES-1 cells proliferation were performed by the WST assay. GES-1 cells were transfected with miR-338-3p mimics, NC mimics, anti-miR-338-3p or anti-NC at a final concentration of 100 nM.The WST assay was performed every 24 h for 4 days. The results are means of three independent experiments ± S.D.(*P<0.05). (B) Colonies were counted. The results were means of three independent experiments ± S.D.(*P<0.05). (C) Representative histograms depicting apoptosis of GES-1 cells transfected with miR-338-3p mimics, NC mimics, anti-miR-338-3p or anti-NC. Cells were stained with PI and Annexin V-FITC at 48 h post-transfection. (D) The percentage of apoptotic cells of three independent experiments ± S.D. are shown (**P<0.01). (E) Representative photographs of migratory cells on the membrane (magnification 100×). None of the GES-1 cells have the migration nature of cancer cells. **Figure S2. Inhibition of miR-338-3p induces the proliferation of SGC-7901 cells.** (A) SGC-7901 cells proliferation were performed by the WST assay. SGC-7901 cells were transfected with anti-miR-338-3p or anti-NC at a final concentration of 100 nM.The WST assay was performed every 24 h for 4 days. The results are means of three independent experiments ± S.D.(*P<0.05). (B) Representative photographs of colonies. (C) Colonies were counted. The results were means of three independent experiments ± S.D. (*P<0.05). **Figure S3. Inhibition of miR-338-3p induces migration and invasion of SGC-7901 cells.** (A) Representative photographs of migratory cells on the membrane (magnification 100×).(B) Average migratory cell number of three independent experiments ± S.D. (*P<0.05). (C) Representative photographs of invasive cells on the membrane (magnification 100×). (D) Average invasive cell number of three independent experiments ± S.D. (*P<0.05). **Figure S4. Inhibition of miR-338-3p inhibits apoptosis of SGC-7901 cells.** (A–B) Representative histograms depicting apoptosis of SGC-7901 cells transiently transfected with anti-miR-338-3p or anti-NC. Cells were stained with PI and Annexin V-FITC at 48 h post-transfection. (C) The percentage of apoptotic cells of three independent experiments ± S.D. are shown (*P<0.05). **Figure S5. SSX2IP has no influence on the expression of miR-338-3p.** Both SGC-7901 cells and GES-1 cells were transiently transfected with GFP-SSX2IP, GFP-Vector control, siRNA-SSX2IP or siRNA-NC. Relative expressions of miR-338-3p in these cell lines were detected by qPCR.
(DOC)
