# Supplementary figures and images for: Epigenetic Silencing of miR-338-3p Contributes to Tumorigenicity in Gastric Cancer by Targeting SSX2IP
Source: PLoS One. 2013 Jun 24;8(6):e66782. doi: 10.1371/journal.pone.0066782 (PMC3691322; doi:10.1371/journal.pone.0066782)

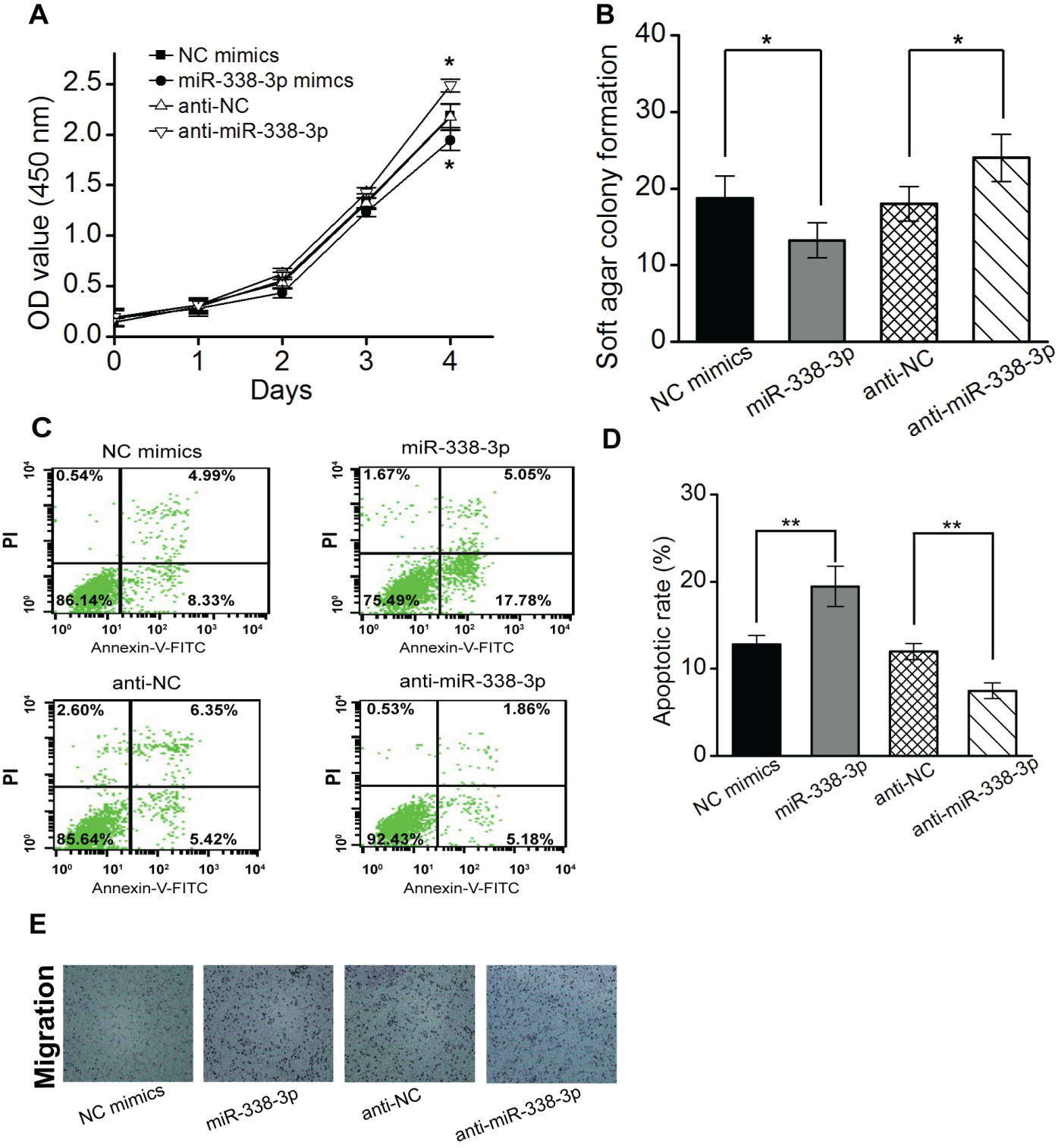


Figure S1


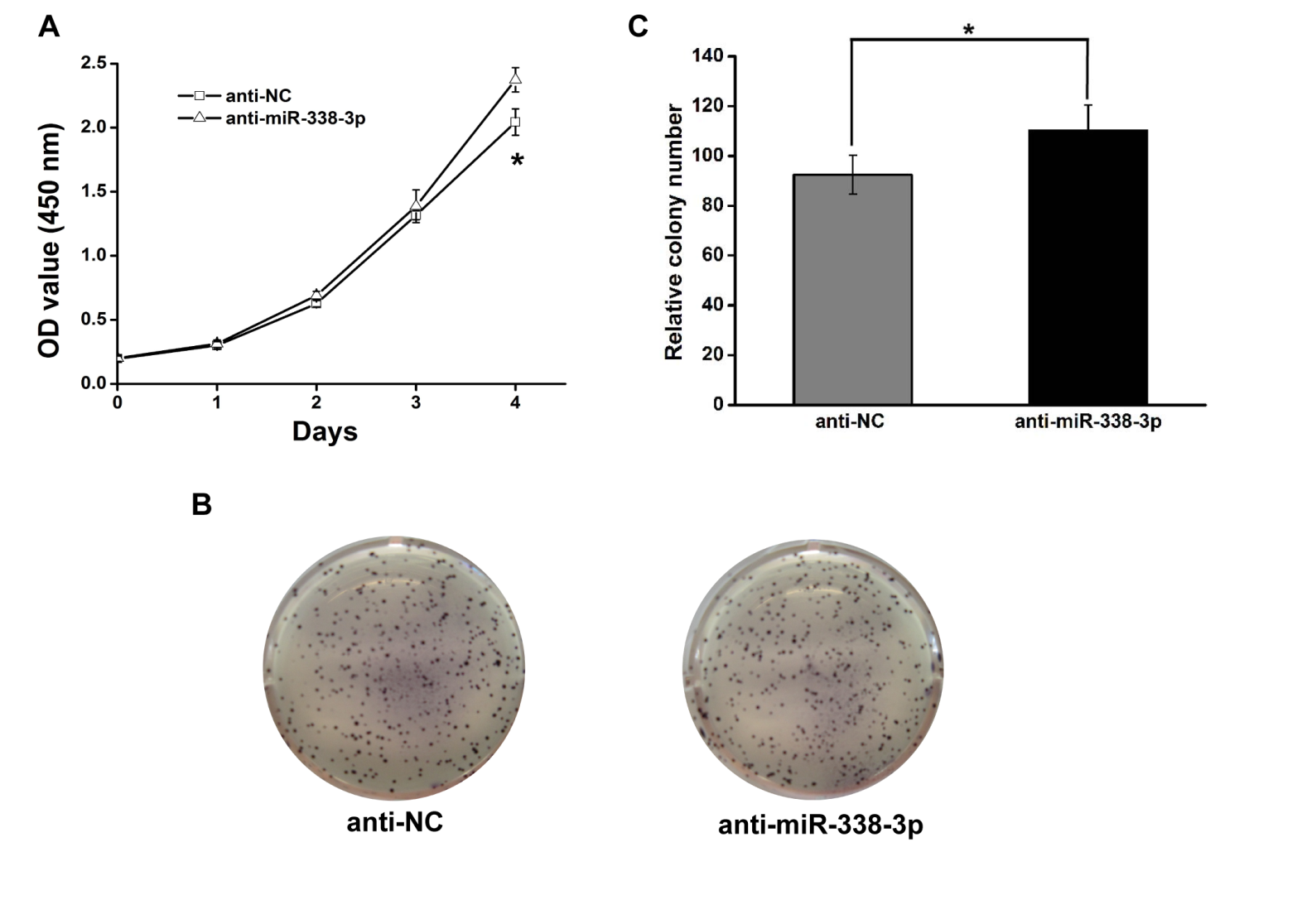


Figure S2


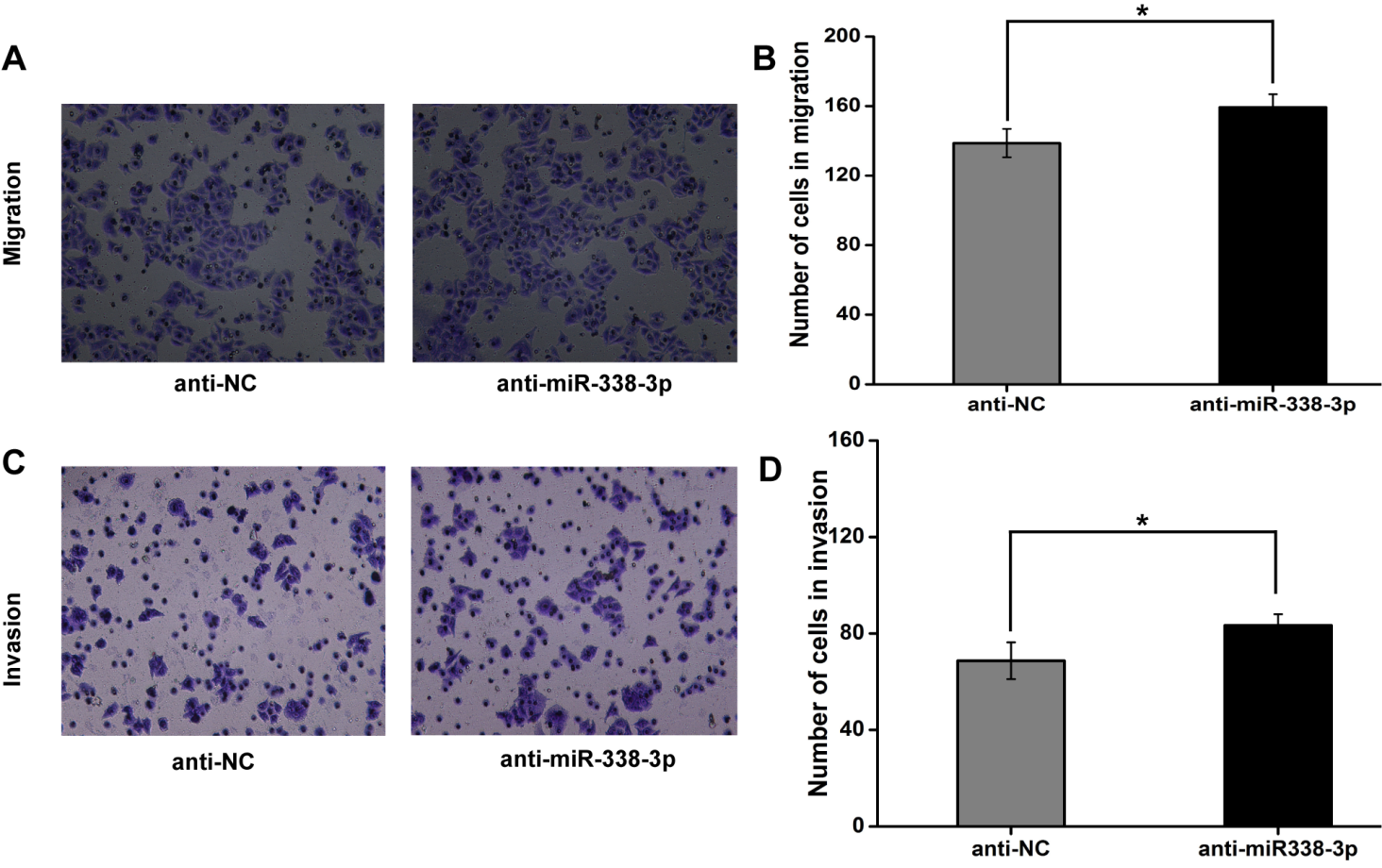


Figure S3


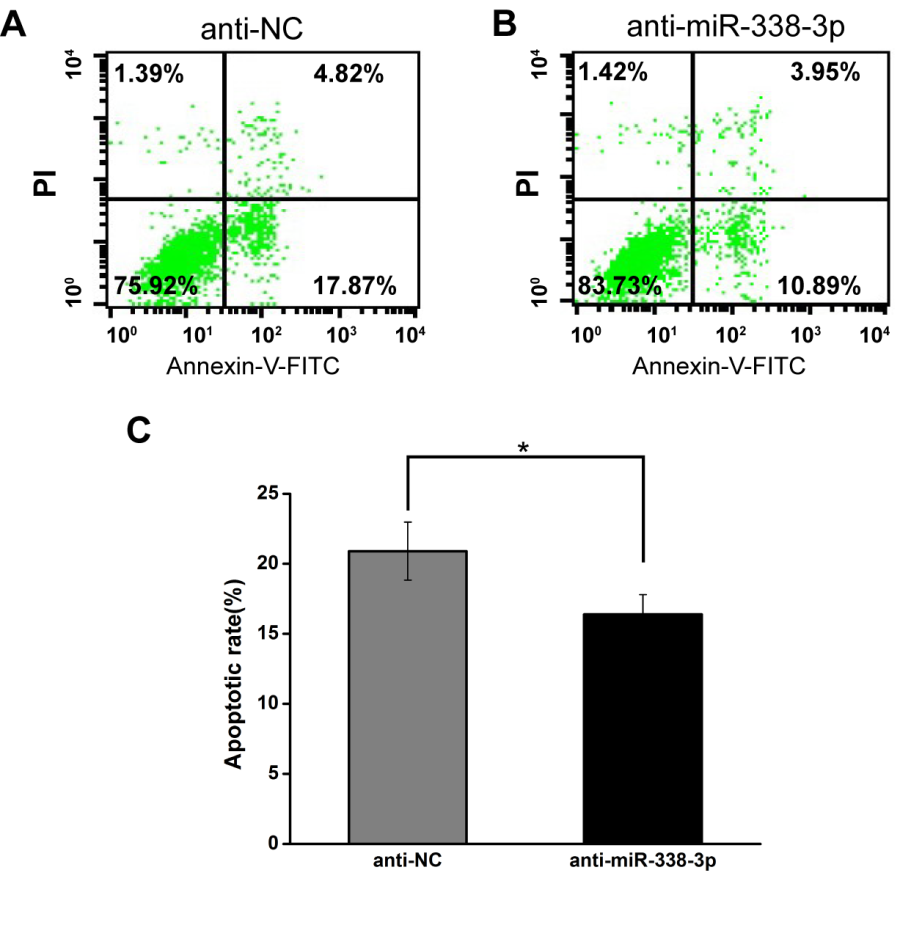


Figure S4


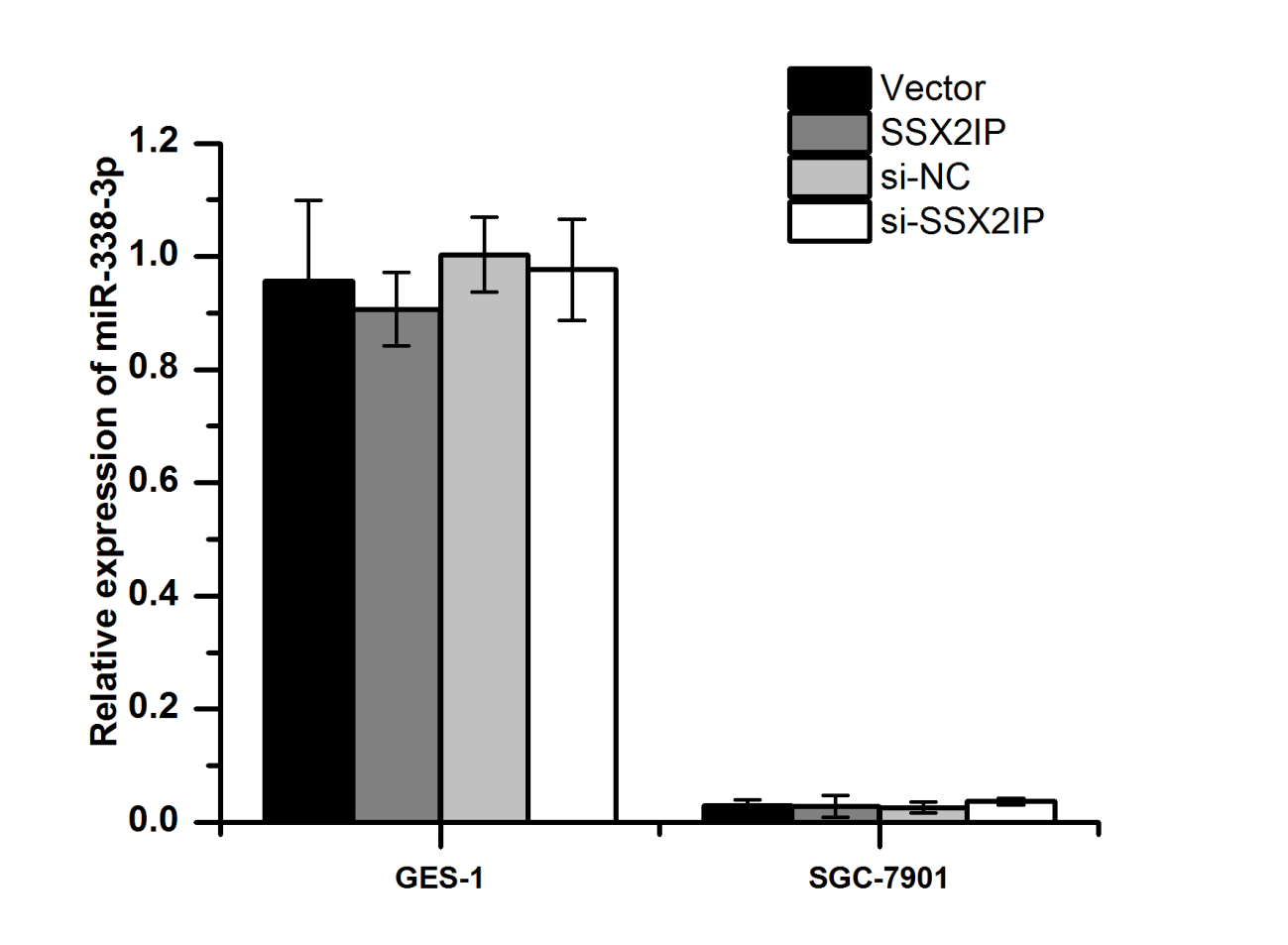


Figure S5

Supplement: File S1 — Supporting information figures. Figure S1. miR-338-3p inhibits GES-1 proliferation and induces apoptosis, but has no influence on migration. (A) GES-1 cells proliferation were performed by the WST assay. GES-1 cells were transfected with miR-338-3p mimics, NC mimics, anti-miR-338-3p or anti-NC at a final concentration of 100 nM.The WST assay was performed every 24 h for 4 days. The results are means of three independent experiments ± S.D.(*P<0.05). (B) Colonies were counted. The results were means of three independent experiments ± S.D.(*P<0.05). (C) Representative histograms depicting apoptosis of GES-1 cells transfected with miR-338-3p mimics, NC mimics, anti-miR-338-3p or anti-NC. Cells were stained with PI and Annexin V-FITC at 48 h post-transfection. (D) The percentage of apoptotic cells of three independent experiments ± S.D. are shown (**P<0.01). (E) Representative photographs of migratory cells on the membrane (magnification 100×). None of the GES-1 cells have the migration nature of cancer cells. Figure S2. Inhibition of miR-338-3p induces the proliferation of SGC-7901 cells. (A) SGC-7901 cells proliferation were performed by the WST assay. SGC-7901 cells were transfected with anti-miR-338-3p or anti-NC at a final concentration of 100 nM.The WST assay was performed every 24 h for 4 days. The results are means of three independent experiments ± S.D.(*P<0.05). (B) Representative photographs of colonies. (C) Colonies were counted. The results were means of three independent experiments ± S.D. (*P<0.05). Figure S3. Inhibition of miR-338-3p induces migration and invasion of SGC-7901 cells. (A) Representative photographs of migratory cells on the membrane (magnification 100×).(B) Average migratory cell number of three independent experiments ± S.D. (*P<0.05). (C) Representative photographs of invasive cells on the membrane (magnification 100×). (D) Average invasive cell number of three independent experiments ± S.D. (*P<0.05). Figure S4. Inhibition of miR-338-3p [file pone.0066782.s001.doc]
